# Synergistic Activations of *REG I***α**** and *REG I* ***β*** Promoters by IL-6 and Glucocorticoids through JAK/STAT Pathway in Human Pancreatic ***β*** Cells

**DOI:** 10.1155/2015/173058

**Published:** 2015-02-12

**Authors:** Akiyo Yamauchi, Asako Itaya-Hironaka, Sumiyo Sakuramoto-Tsuchida, Maiko Takeda, Kiyomi Yoshimoto, Tomoko Miyaoka, Takanori Fujimura, Hiroki Tsujinaka, Chikatsugu Tsuchida, Hiroyo Ota, Shin Takasawa

**Affiliations:** Department of Biochemistry, Nara Medical University, Kashihara 634-8521, Japan

## Abstract

*Reg* (*Regenerating gene*) gene was originally isolated from rat regenerating islets and its encoding protein was revealed as an autocrine/paracrine growth factor for *β* cells. Rat *Reg* gene is activated in inflammatory conditions for *β* cell regeneration. In human, although five functional *REG* family genes (*REG Iα*, REG I*β*, *REG III, HIP/PAP*, and *REG IV*) were isolated, their expressions in *β* cells under inflammatory conditions remained unclear. In this study, we found that combined addition of IL-6 and dexamethasone (Dx) induced *REG Iα* and *REG Iβ* expression in human 1.1B4 *β* cells. Promoter assay revealed that a signal transducer and activator of transcription- (STAT-) binding site in each promoter of *REG Iα* (TGCCGGGAA) and *REG Iβ* (TGCCAGGAA) was essential for the IL-6+Dx-induced promoter activation. A Janus kinase 2 (JAK2) inhibitor significantly inhibited the IL-6+Dx-induced *REG Iα* and *REG Iβ* transcription. Electrophoretic mobility shift assay and chromatin immunoprecipitation revealed that IL-6+Dx stimulation increased STAT3 binding to the *REG Iα* promoter. Furthermore, small interfering RNA-mediated targeting of STAT3 blocked the IL-6+Dx-induced expression of *REG Iα* and *REG Iβ*. These results indicate that the expression of *REG Iα* and *REG Iβ* should be upregulated in human *β* cells under inflammatory conditions through the JAK/STAT pathway.

## 1. Introduction

Decreased functional *β* cell mass is one of hallmarks in both type 1 and type 2 diabetes. Regeneration of pancreatic *β* cells has been shown to occur at a basal rate in normal adult tissues and to increase under certain conditions such as pregnancy and obesity [1–3]. However, the mechanism of human pancreatic *β* cell regeneration still remains to be elucidated.

*Reg* (*Regenerating gene*) gene product, Reg protein, could be responsible for the regenerative process [4–6]. The* Reg* gene was originally isolated from regenerating pancreatic islets from depancreatized rats, and it encodes a 16 kDa autocrine/paracrine growth factor for *β* cells [7, 8]. The* Reg* and* Reg*-related genes were isolated and revealed to constitute a multigene family, the* Reg* family, which consists of four subtypes (types I, II, III, and IV) based on the primary structures of the encoded proteins of the genes [4, 5, 9, 10]. The type I* Reg* gene,* Reg I*, was the first isolated* Reg* gene from the regenerating islets [7]. In rat and mouse, Reg I protein is not expressed in pancreatic *β* cells in physiological conditions but is induced during islet regeneration [4, 7, 11, 12]. Therefore, the* Reg I* gene induction, which occurs in response to inflammatory mediators such as interleukin- (IL-) 6 and glucocorticoid analogue dexamethasone (Dx) in rat RINm5F *β* cells [13], is considered to be one of the crucial events in *β* cell regeneration. In human, five functional* REG* family genes (*REG Iα*,* REG Iβ*,* REG III*,* HIP/PAP*, and* REG IV*) were isolated [4, 7, 14–19]. However, which human* REG* gene is expressed in *β* cells during regeneration is still obscure mainly because of their restrict supply of human islets. Induction of proliferation/regeneration in human *β* cells must be beneficial for both type 1 and type 2 diabetes treatment/prevention [20]. The human* REG* genes would play an important role in *β* cell proliferation/regeneration in human as the* Reg I* gene does in rodents. Since there are many differences in cell proliferation between rodent and human *β* cells [21], it is crucial to investigate the expression of human* REG* genes in human *β* cells. Recently, *β* cell line, 1.1B4, was established and becomes available as human pancreatic *β* cells [22]. In the present study, we investigated the induction of human* REG* family genes in inflammatory conditions in 1.1B4 human *β* cells.

## 2. Materials and Methods

### 2.1. Cell Culture and Reagents

Human 1.1B4 cells, an insulin-releasing human pancreatic *β* cell line (European Collection of Cell Culture, Salisbury, UK) [22, 23], and rat RINm5F cells were grown in RPMI 1640 medium (NACALAI TESQUE, Kyoto, Japan) containing 10% (v/v) fetal calf serum, 100 units/mL penicillin G, and 100 *μ*g/mL streptomycin (Wako Pure Chemical Industries, Osaka, Japan) as described [23]. The cells were maintained in a 5% CO_2_-95% air, water-saturated atmosphere at 37°C. Recombinant human IL-6, tumor necrosis factor- (TNF-) *α*, and interferon- (IFN-) *γ* were purchased from Roche Applied Science (Indianapolis, IN). Recombinant human IL-1*β*, IL-22, IFN-*β*, IL-8, and H_2_O_2_ were obtained from Wako Pure Chemical Industries. Recombinant human platelet-derived growth factor (PDGF) was obtained from PeproTech (Rocky Hill, NJ). Sodium palmitate (Sigma, St. Louis, MO) was prepared as albumin-bound form by stirring at 45°C with defatted bovine serum albumin (Cosmo Bio Co., Tokyo, Japan). Dexamethasone (Dx) was purchased from MP Biomedicals (Santa Ana, CA).* S*-Nitroso-*N*-acetylpenicillamine (SNAP), a NO donor [24], and AG490, a Janus kinase 2 (JAK2) inhibitor [25], were obtained from Calbiochem (Billerica, MA) and were dissolved in dimethyl sulfoxide as stock solutions. Streptozotocin (STZ) (Sigma) was dissolved in citrate buffer (pH 4.5) and further diluted with cell culture media just before use. 3-Aminobenzamide (3AB) was purchased from Tokyo Kasei Co. (Tokyo, Japan).

### 2.2. Promoter Assays

The reporter constructs were prepared by inserting the 5′-flanking regions of human* REG* family genes (*REG Iα*, GenBank Accession No. J05412; −1190~+26, −569~+26, −462~+26, −168~+26, −146~+26, −130~+26,* REG Iβ*, GenBank Accession No. D17291; −978~+31, −155~+31, −139~+31,* REG III*, GenBank Accession No. AB161039; −1550~+64,* HIP/PAP*, GenBank Accession No. X79987; −1520~+17,* REG IV*, GenBank Accession No. AL592186; −1053~+22) upstream of a firefly luciferase reporter gene in pGL3-Basic vector (Promega, Madison, WI). For construction of the promoter region mutants used in this study, the indicated sequences in the “−146”* REG Iα*/luciferase constructs and “−155”* REG Iβ*/luciferase constructs were substituted by primers using PCR. The cells were grown in 24-well plates and were transfected with reporter plasmids by lipofection. Briefly, 0.8 *μ*g of each reporter plasmid and 0.08 *μ*g of pCMV-SPORT-*β*-galactosidase (Life Technologies, Carlsbad, CA), as an internal control, were mixed with Lipofectamine 2000 (Life Technologies) per well in a 24-well plate. After 24 h incubation, the cells were treated with indicated amount of stimulants, 100 nM Dx, 20 ng/mL IL-6, 300 U/mL IL-1*β*, 370 U/mL TNF-*α*, 100 U/mL IFN-*γ*, 60 U/mL IL-1*β* + 185 U/mL TNF-*α* + 14 U/mL IFN-*γ*, 10 ng/mL IL-22, 1500 U/mL IFN-*β*, 100 nM IL-8, and 1 U/mL PDGF, amino acids at a concentration five times greater than that in basal RPMI, 100 *μ*M palmitate, 10 *μ*M H_2_O_2_, 250 *μ*M SNAP, 2 mM STZ, and 2 mM 3AB, and combinations thereof. After 24 h treatment, the cells were harvested and processed for luciferase assay as described [13, 26]. In cases of STZ and STZ + Dx, the media containing STZ or STZ + Dx were changed with fresh media after 2 h treatment, and cells were further incubated for 22 h before harvest.

### 2.3. Quantitative Real-Time RT-PCR

Total RNA was isolated using the RNeasy Plus Mini Kit (Qiagen, Hilden, Germany) from 1.1B4 cells, and cDNA was synthesized from total RNA as a template using High Capacity cDNA Reverse Transcription Kit (Applied Biosystems, Foster City, CA) for the template of real-time PCR as described [23, 27, 28]. The cDNA was subjected to PCR with the following primers: *β-actin* (NM_001101) sense, 5′-GCGAGAAGATGACCCAGA-3′ and antisense, 5′- CAGAGGCGTACAGGGATA-3′;* REG Iα* (NM_002909) sense, 5′-AGGAGAGTGGCACTGATGACTT-3′ and antisense, 5′-TAGGAGACCAGGGACCCACTG-3′;* REG Iβ* (NM_006507) sense 5′-GCTGATCTCCTCCCTGATGTTC-3′ and antisense 5′-GGCAGCTGATTCGGGGATTA-3′;* REG III* (AB161037) sense 5′-GAATATTCTCCCCAAACTG-3′ and antisense 5′-GAGAAAAGCCTGAAATGAAG-3′;* HIP/PAP* (NM_138937) sense 5′-AGAGAATATTCGCTTAATTCC-3′ and antisense 5′-AATGAAGAGACTGAAATGACA-3′;* REG IV* (AY007243) sense 5′-ATCCTGGTCTGGCAAGTC-3′ and antisense 5′-CGTTGCTGCTCCAAGTTA-3′. All the PCR primers were synthesized by Nihon Gene Research Laboratories (Sendai, Japan). Real-time PCR was performed using KAPA SYBR FAST qPCR Master Mix (Kapa Biosystems, Boston, MA) and the Thermal Cycler Dice Real-Time System (Takara, Otsu, Japan) as described [23, 27, 28]. PCR was performed with an initial step of 3 min at 95°C followed by 40 cycles of 3 s at 95°C and 20 s at 60°C for *β-actin*,* REG III*, and* HIP/PAP* and 40 cycles of 3 s at 95°C and 20 s at 64°C for* REG Iα, REG Iβ*, and* REG IV*. The level of target mRNA was normalized to the mRNA level of *β*-*actin* as an internal standard.

### 2.4. Electrophoretic Mobility Shift Assay (EMSA)

Nuclear extracts were prepared as described previously and stored at −80°C until use [13, 29]. EMSAs were performed essentially as described previously [13]. DNA probes for EMSAs were synthesized as oligonucleotides. The sequences of the individual oligonucleotides in the sense orientation were as follows: probe 1, 5′-AGTGTGTGCCGGGAAAAGGCTCATA-3′ (nt. −148~−124 of human* REG Iα* promoter; nt. 1048–1072 of J05412), probe M1, 5′-AGTGTGCAGTAGGAAAAGGCTCATA-3′.

DNA-protein binding reactions were performed by incubation of the nuclear extracts in a solution containing 10 mM HEPES (pH 7.8), 50 mM KCl, 5 mM MgCl_2_, 1 mM EDTA, 10% glycerol, 5 mM DTT, 1 mg/mL BSA, 0.7 mM PMSF, 50 ng/*μ*L of poly(dI-dC) (Roche) for 10 min at room temperature, followed by an additional 30 min incubation with ^32^P-end-labeled probe at room temperature. For supershift assays, 1 *μ*g of anti-signal transducer and activator of transcription (STAT)3 antibody (Santa Cruz Biotechnology, SC-482X, Santa Cruz, CA) [30] was added to the samples and incubated for 15 min before incubation with the labeled probe. DNA-protein complexes were separated on 4% nondenaturing acrylamide gels and detected by autoradiography.

### 2.5. Chromatin Immunoprecipitation (ChIP) Assay

ChIP assay was performed using a ChIP-IT Express kit (Active Motif, Carlsbad, CA) following the manufacturer's instructions as described previously [31]. Briefly, 1.1B4 cells were treated with or without IL-6 + Dx and cross-linked with 1% formaldehyde for 10 min at room temperature. After washing and treatment with glycine Stop-Fix solution, the cells were lysed and nuclei were subjected to ultrasonic disruption for preparing DNA fragments ranging in size from 200 to 1500 bp. Immunoprecipitation was carried out overnight at 4°C using an incubation mixture containing sheared chromatin, protein G magnetic beads, and 3 *μ*g of anti-STAT3 antibody (Santa Cruz Biotechnology, SC-482X). The immune complexes were precipitated, eluted, reverse cross-linked, and treated with proteinase K. The resulting DNA samples were subjected to PCR amplification (1 cycle of 94°C for 3 min, 35 cycles of 94°C for 30 s, 62°C for 30 s, 72°C for 1 min) of the* REG Iα* promoter using specific forward (5′-ACCTTGGACTTAGACAGCTTG-3′) and reverse (5′-ACCACGTCATTTAAGCAAAAGG-3′) primers designed to amplify the promoter region (−212 to −46 nucleotides in relation to the transcription start site). The PCR products were resolved by electrophoresis in a 2.5% agarose gel containing ethidium bromide for visualization.

### 2.6. RNA Interference (RNAi)

RNAi was performed using* Silencer* Select predesigned small interfering RNAs (siRNAs) (Life Technologies) [23] directed against human* STAT3* gene. The sense sequence of siRNA (5′-GCACCUUCCUGCUAAGAUUtt-3′) was synthesized by Nihon Gene Research Laboratories. As a control, siRNA-scramble (Ambion, Life Technologies) [23] was also used. Transfection of siRNA to 1.1B4 cells was carried out using Lipofectamine RNAiMAX (Life Technologies). Cells were transfected with 5 pmol of siRNA in a 24-well culture dish (4 × 10^5^ cells/mL) as described [23].

### 2.7. Data Analysis

Results are expressed as mean ± SE. Statistical significance was determined by Student's *t*-test using GraphPad Prism software (GraphPad Software, La Jolla, CA).

## 3. Results

### 3.1. Activation of Human* REG* Family Gene Promoters in *β* Cells

**Figure 1:**
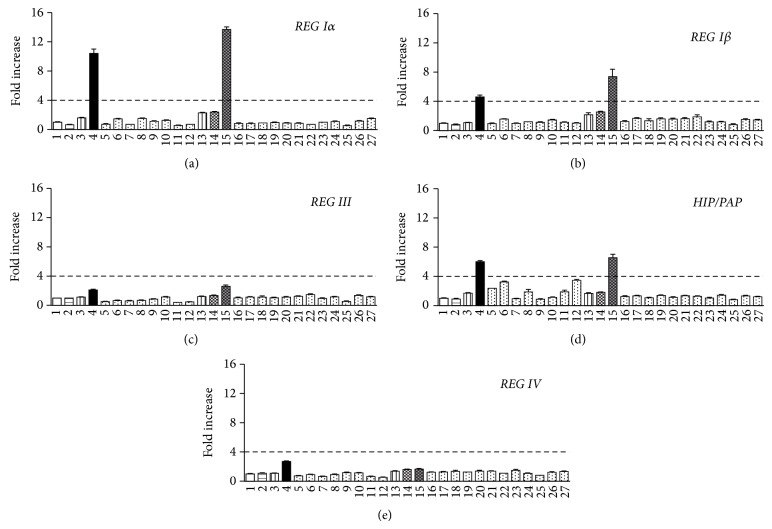
Effects of cytokines, *β*-cell toxic agents, and growth factors on the transcription of (a)* REG Iα*, (b)* REG Iβ*, (c)* REG III,* (d)* HIP/PAP*, and (e)* REG IV* in human 1.1B4 *β* cells. Cells were transfected with the human* REG* family gene reporter plasmids and treated as follows: 1, No addition; 2, Dx (100 nM); 3, IL-6 (20 ng/mL); 4, IL-6 + Dx; 5, IL-1*β* (300 U/mL); 6, IL-1*β* + Dx; 7, TNF-*α* (370 U/mL); 8, TNF-*α* + Dx; 9, IFN-*γ* (100 U/mL); 10, IFN-*γ* + Dx; 11, IL-1*β* (60 U/mL) + TNF-*α* (185 U/mL) + IFN-*γ* (14 U/mL); 12, IL-1*β* + TNF-*α* + IFN-*γ* + Dx; 13, IL-22 (10 ng/mL); 14, IL-22 + Dx; 15, IL-22 + IL-6 + Dx; 16, IFN-*β* (1500 U/mL); 17, IFN-*β* + Dx; 18, IL-8 (100 nM); 19, IL-8 + Dx; 20, PDGF (1 U/mL); 21, PDGF + Dx; 22, amino acids (5 times greater than that in RPMI 1640 medium); 23, palmitate (100 *μ*M); 24, H_2_O_2_ (10 *μ*M); 25, SNAP (250 *μ*M); 26, STZ (2 mM); 27, STZ + Dx. The promoter activity was normalized for variations in transfection efficiency using *β*-galactosidase activity as an internal standard. Fold increase is calculated by dividing the promoter activity of stimulated cells by that of unstimulated cells (column 1). The broken line corresponds to 4-fold increase. Data are expressed as means ± SE for each group ((a) *N* = 3-4, (b–e) *N* = 3). Transcriptional activities of no. 4 and 15 (IL-6 + Dx and IL-22 + IL-6 + Dx) in panels (a), (b), and (d) were significantly (*P* < 0.005) and prominently (over 4 fold) increased.

We first tested various stimuli on human* REG* family gene transcription in human 1.1B4 *β* cells. As* Reg* gene expression was observed in the phase of transient *β* cell proliferation: for example, in pancreatic islets of diabetic BB/Wor//Tky rats during the remission phase of diabetes [32], in islets of nonobese diabetic mice during active diabetogenesis [33] and pancreatic ductal cells, which are thought to be progenitor cells of *β* cells, during differentiation and proliferation in a mouse model of autoimmune diabetes [34, 35], and inflammation and cell death in and/or around islets was involved in these cases, we used inflammatory cytokines such as IL-1*β*, TNF-*α*, and IFN-*γ* [36], as well as pancreatic *β* cell toxic agents, such as palmitate [37, 38], H_2_O_2_ [39, 40], SNAP [41, 42], and STZ [43, 44]. Other factors, which were reported to upregulate* Reg* gene expression in *β* cells, such as IL-6 [13, 31], glucocorticoid [13, 31], IL-22 [6, 45], IFN-*β* [35], PDGF [46], and amino acids [46], were tested. We also used IL-8, which was reported to stimulate* REG Iα* promoter activity in gastric endocrine carcinoma cells [47]. As shown in Figure 1(a), IL-6 and IL-22 modestly increased the* REG Iα* promoter activity (columns 3 and 13). When IL-6 and the glucocorticoid analogue Dx were added together, the promoter activity was remarkably increased (columns 3 and 4). In contrast, the combination of IL-22 and Dx did not further increase the promoter activity (columns 13 and 14). The combination of IL-22 and IL-6 + Dx did not result in the synergistic response (columns 14 and 15). Treatments with other stimulants were ineffective or resulted in only small changes in promoter activity of* REG Iα* gene compared to IL-6 + Dx. These results showed that IL-6 + Dx was the most effective inducer for human* REG Iα* gene transcription in human *β* cells. IL-6 + Dx also enhanced the promoter activities of* REG Iβ* and* HIP/PAP* significantly; however, the fold increases were much smaller compared to that of* REG Iα* (Figures 1(a), 1(b), and 1(d)). Although the transcription of* REG III* and* REG IV* was induced to some extent by IL-6 + Dx, the increments were quite small (Figures 1(c) and 1(e)). The other treatments did not induce* REG Iα*,* REG Iβ*,* REG III*,* HIP/PAP*, nor* REG IV* transcription as much as IL-6 + Dx did.

**Figure 2:**
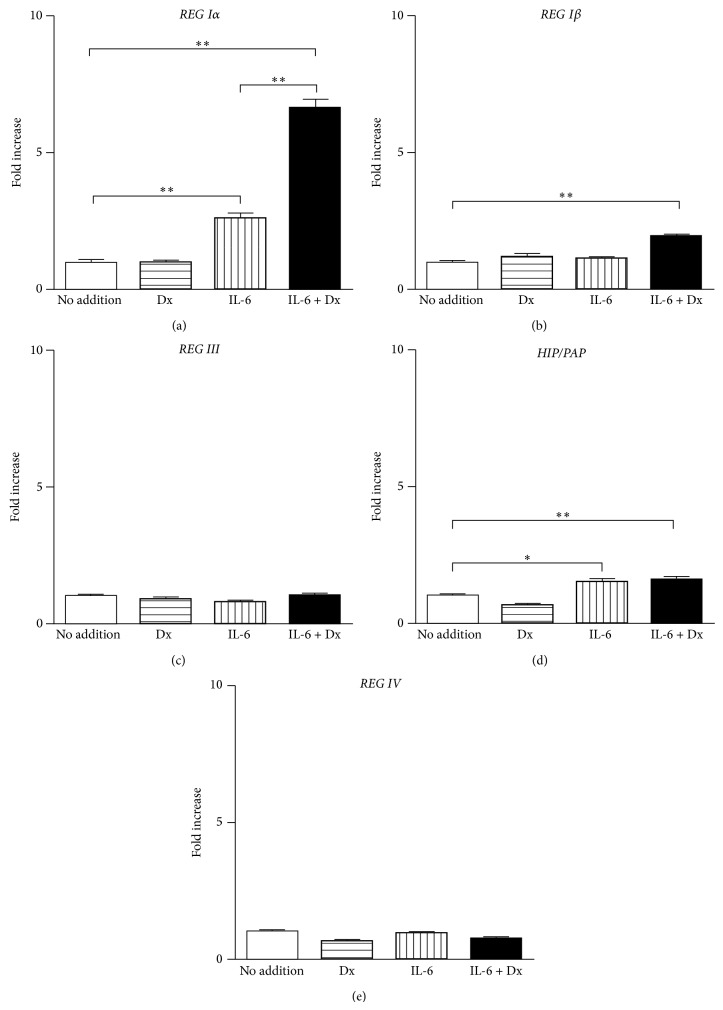
Effects of Dx and IL-6 on the promoter activity of (a)* REG Iα*, (b)* REG Iβ*, (c)* REG III*, (d)* HIP/PAP*, and (e)* REG IV* in rat RINm5F cells. RINm5F cells were transfected with the human* REG* family gene reporter plasmids and treated without (no addition) or with Dx (100 nM), or IL-6 (20 ng/mL), or IL-6 + Dx. The promoter activity was normalized for variations in transfection efficiency using *β*-galactosidase activity as an internal standard. Fold increase is calculated by dividing the promoter activity of stimulated cells by that of unstimulated cells (No addition). Data are expressed as means ± SE for each group (*N* = 3). ^*^ *P* < 0.01; ^**^ *P* < 0.005.

To determine whether the IL-6 + Dx-induced expression of* REG* family gene was restricted only in 1.1B4 cells, we investigated the effects of IL-6 + Dx on the promoter activities of human* REG* genes in rat RINm5F *β* cells. Similarly in 1.1B4 cells, the transcription of* REG Iα* was synergistically increased by the combined addition of IL-6 and Dx (Figure 2(a)). The* REG Iβ* transcription was also significantly induced by IL-6 + Dx in RINm5F cells (Figure 2(b)); however, the increase was not so large as that in 1.1B4 cells. The transcriptional activity of* HIP/PAP* was also increased by IL-6 and IL-6 + Dx about 1.5-fold (Figure 2(d)). On the other hand, the transcriptional activities of* REG III* and* REG IV* were never induced by Dx, IL-6, nor IL-6 + Dx (Figures 2(c) and 2(e)). These results indicated that activation of human type I* REG* gene transcription by IL-6 + Dx is general feature in pancreatic *β* cells.

### 3.2. Induction of mRNAs for* REG Iα* and* REG Iβ* by IL-6 + Dx in *β* Cells

**Figure 3:**
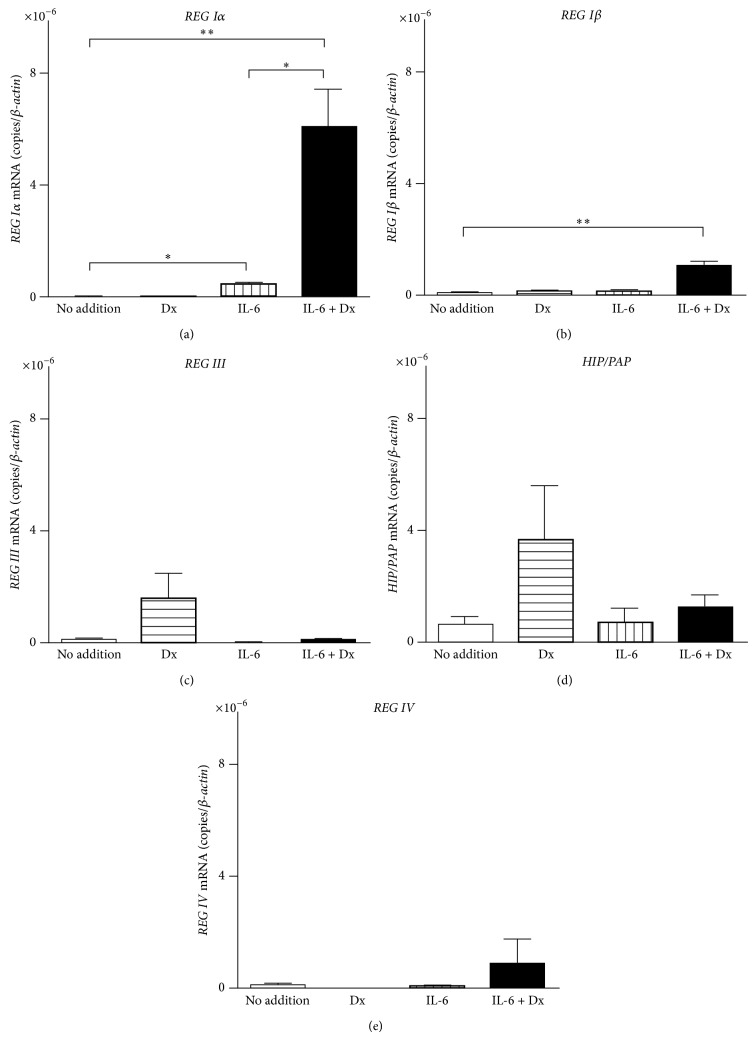
The mRNA levels of (a)* REG Iα*, (b)* REG Iβ*, (c)* REG III*, (d)* HIP/PAP*, and (e)* REG IV* in human 1.1B4 cells treated without (no addition) or with Dx (100 nM), or IL-6 (20 ng/mL), or IL-6 + Dx. Data are represented as the ratio of the number of target mRNA copies to the number of *β-actin* mRNA copies and expressed as means ± SE for each group (*N* = 4). ^*^ *P* < 0.05; ^**^ *P* < 0.005.

We next analyzed the mRNA levels of intrinsic human* REG* family genes in 1.1B4 cells treated with IL-6 and Dx by real-time RT-PCR. The mRNA levels of* REG Iα* and* REG Iβ* were significantly increased by the addition of IL-6 + Dx (Figures 3(a) and 3(b)). On the other hand, the mRNA levels of* REG III*,* HIP/PAP*, and* REG IV* were not significantly increased by the addition of neither IL-6, Dx, nor IL-6 + Dx (Figures 3(c), 3(d) and 3(e)). These results strongly suggest that type I* REG* family genes (*REG Iα* and* REG Iβ*) were induced in human *β* cells under inflammatory conditions by increased levels of circulating IL-6 and glucocorticoids.

### 3.3. IL-6 + Dx Response Element in* REG* Gene Promoter

**Figure 4:**
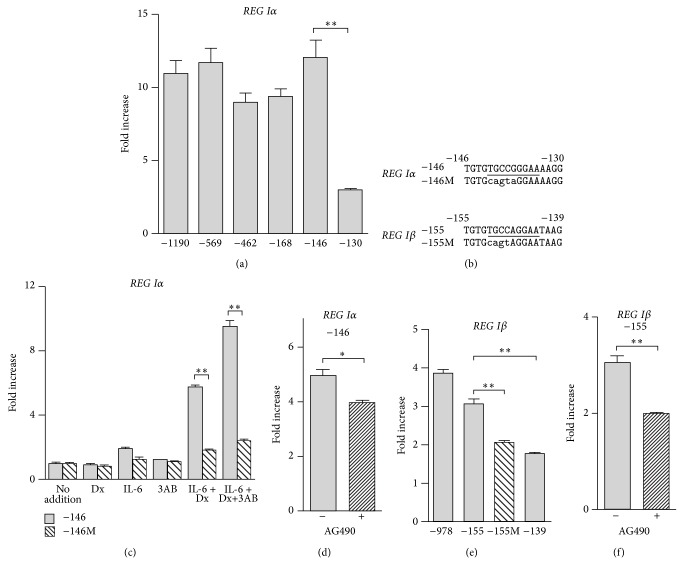
IL-6 + Dx-induced transcription of* REG Iα* and* REG Iβ* in human 1.1B4 *β* cells is dependent on the JAK/STAT pathway. (a) IL-6 + Dx-induced* REG Iα* promoter activation in 1.1B4 cells. (b) Sequences of the human* REG Iα* and* REG Iβ* promoters. Putative STAT-binding site is underlined. Nucleotide substitutions in the STAT-binding site are indicated by lower-case letters. (c) Effects of site-directed mutation on IL-6 + Dx-induced* REG Iα* promoter activation. (d) Effects of AG490, a JAK inhibitor, on IL-6 + Dx-induced* REG Iα* promoter activity. 1.1B4 cells were transfected with indicated construct. (e) IL-6 + Dx-induced* REG Iβ* promoter activation in deleted and mutated promoter. (f) Effects of JAK inhibitor on IL-6 + Dx-induced* REG Iβ* promoter activity. Fold increase is calculated by dividing the promoter activity of IL-6 + Dx treated cells by that of untreated cells. Data are expressed as means ± SE for each group ((a) *N* = 4, (c) *N* = 3-4, (d) *N* = 3, (e) *N* = 3-4, (f) *N* = 4). ^*^ *P* < 0.05; ^**^ *P* < 0.005.

To identify the region essential for* REG Iα* induction by IL-6 + Dx, the reporter plasmids containing progressively deleted promoter fragments of* REG Iα* (−1190~+26, −569~+26, −462~+26, −168~+26, −146~+26, −130~+26) upstream of luciferase gene were transfected into 1.1B4 cells. Deletion of the* REG Iα* promoter from position of −1190 to −146 retained a significant increase in response to IL-6 + Dx in luciferase activity (Figure 4(a)). In contrast, further deletion to position −130 caused the loss of inducibility. These data suggest that the region between −146 and −131 is essential for the IL-6 + Dx-induced* REG Iα* transcription. The sequence analysis revealed the presence of elements homologous to the STAT-binding site between position −142 and −134 (TGCCGGGAA) (Figure 4(b)). Site-directed mutagenesis was conducted within the luciferase construct “−146,” and the influence of the mutation (“−146 M” in Figure 4(b)) on the amplitude of luciferase induction by IL-6 + Dx was monitored. The replacement of the STAT-binding sequence to the sequence CAGTAGGAA, which disrupted the binding of STAT (“−146 M”), significantly blocked the IL-6 + Dx-induced transcriptional activation of* REG Iα* in 1.1B4 cells (Figure 4(c)).

In rat RINm5F cells, the cis-element of rat* Reg I* gene for induction in response to IL-6 + Dx was the −81~−70 region, which corresponds to the −76~−65 region of human* REG Iα* gene [13]. In RINm5F cells, poly(ADP-ribose) polymerase (PARP) was found to bind to the cis-element of* Reg I* gene and was involved in the active transcriptional DNA/protein complex formed by the stimulation of IL-6 + Dx. The DNA/protein complex formation was inhibited by the autopoly(ADP-ribosyl)ation of PARP in the complex. Thus, PARP inhibitors, such as 3AB, enhanced the DNA/protein complex formation for* Reg I* gene transcription [13]. To clarify whether PARP was involved in the IL-6 + Dx-induced human* REG Iα* transcription, we treated 1.1B4 cells with IL-6 + Dx + 3AB and measured the promoter activities of the constructs “−146” and “−146 M.” As shown in Figure 4(c), 3AB further enhanced the IL-6 + Dx-induced promoter activity of construct “−146.” On the other hand, the promoter activity of mutant construct “−146 M” was not induced as much as that of “−146” by IL-6 + Dx + 3AB, suggesting that STAT rather than PARP played a major role in the induction of human* REG Iα* transcription by IL-6 + Dx.

STAT proteins are activated by JAKs, and IL-6 induces activation of JAKs [48, 49]. Pretreatment of 1.1B4 cells with a JAK2 inhibitor, AG490 (50 *μ*M), for 4 h significantly inhibited the inducibility of* REG Iα* promoter by IL-6 + Dx (Figure 4(d)). These results suggest that the JAK/STAT signaling pathway is involved in the IL-6 + Dx-induced* REG Iα* gene transcription in *β* cells.

As the nucleotide sequence of promoter region of* REG Iα* and* REG Iβ* is very similar, we searched possible STAT-binding sequence in the* REG Iβ* promoter and found that there is a STAT-binding site between position −151 and −143, which corresponds to the STAT-binding site of* REG Iα* (the −142~−134 region) as shown in Figure 4(b). The deletion and mutational analyses revealed that the STAT-binding site was essential to the IL-6 + Dx-induced* REG Iβ* transcription (Figure 4(e)). Moreover, AG490 almost completely blocked the induction of* REG Iβ* promoter by IL-6 + Dx (Figure 4(f)). Thus, it is suggested that IL-6 + Dx-induced* REG Iβ* transcription also depends on the JAK/STAT signaling pathway.

### 3.4. STAT3 Binding to the* REG Iα* Promoter

**Figure 5:**
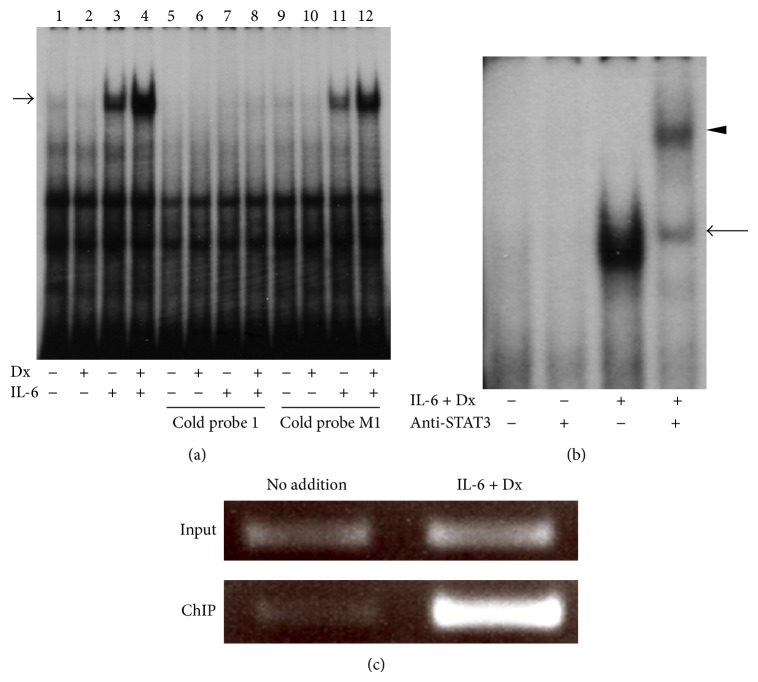
IL-6 + Dx-increased STAT3 binding to the* REG Iα* promoter. (a) Detection of DNA-protein complexes using EMSA. Nuclear extracts (2.5 *μ*g protein) prepared from 1.1B4 cells, treated without or with Dx (100 nM), or IL-6 (20 ng/mL), or IL-6 + Dx, were incubated with a ^32^P-labeled probe 1. Nuclear extracts from untreated cells were applied onto lanes 1, 5, and 9; those from Dx-treated cells were applied onto lanes 2, 6, and 10; those from IL-6-treated cells were applied onto lanes 3, 7, and 11; and those from IL-6 + Dx-treated cells were applied onto lanes 4, 8, and 12. Lanes 1–4 contain no competitor; lanes 5–8 contain 100 × unlabeled probe 1; lanes 9–12 contain 100 × unlabeled probe M1. An arrow marks IL-6 + Dx-inducible complex migration. (b) Supershift assay of STAT3 containing complex. EMSAs were performed in the presence or absence of STAT3 antibodies as indicated. An arrow marks IL-6 + Dx-inducible complex migration. An arrowhead indicates supershift complex by anti-STAT3 antibody. (c) ChIP assay showing IL-6 + Dx increases in STAT3 binding to the* REG Iα* promoter. 1.1B4 cells were treated without or with IL-6 (20 ng/mL) + Dx (100 nM). ChIPs were performed with anti-STAT3 antibody, followed by PCR with primers specific for the* REG Iα* promoter. A representative result from three experiments is shown.

To further investigate whether STAT protein interacts with the* REG Iα* promoter in vitro and in vivo, we performed EMSA and ChIP assays, respectively. Treatment of Dx did not change the nuclear protein binding to the probe 1 containing the STAT-binding sequence (TGCCGGGAA) (Figure 5(a), lane 2). In contrast, treatment of IL-6 resulted in an increase in nuclear protein binding to the probe (lane 3), and the intensity of shift band was further increased by the combined addition of IL-6 and Dx (lane 4). The nuclear protein binding induced by IL-6 + Dx was competed with an excess of unlabeled probe (lane 8). On the other hand, excess amount of unlabeled mutated probe (probe M1), which disrupted the STAT-binding site, could not competitively block the binding to the labeled probe (lane 12). These results showed the binding specificity of the IL-6 + Dx-induced complex. An antibody for STAT3 produced a supershift band (Figure 5(b)), suggesting that STAT3 was involved in this nuclear protein-DNA complex formed by the IL-6 + Dx stimulation. Furthermore, immunoprecipitations of cross-linked DNA-protein complexes derived from IL-6 + Dx-treated 1.1B4 cells with a STAT3 antibody contained the STAT-binding element of the human* REG Iα* gene (Figure 5(c)). These results showed that STAT3 binding to the* REG Iα* promoter was induced by IL-6 + Dx in *β* cells.

### 3.5. STAT3 Is Necessary for the IL-6 + Dx-Induced Type I* REG* Gene Expression

**Figure 6:**
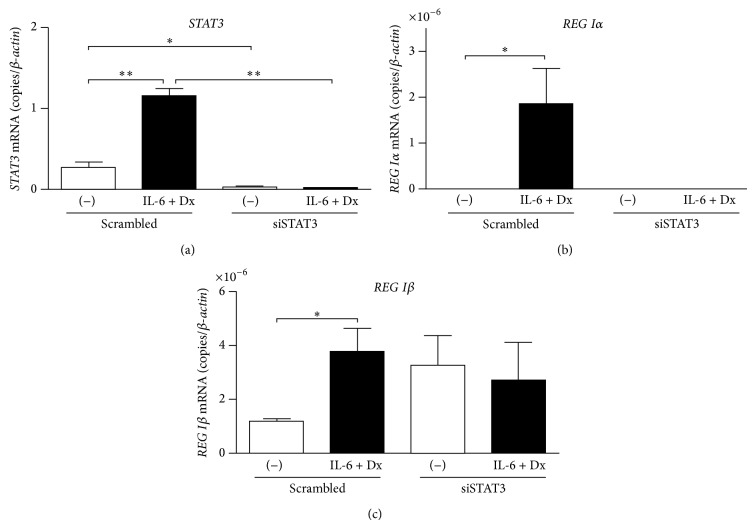
Effects of STAT3 knockdown on IL-6 + Dx-induced mRNA expression of* REG Iα* and* REG Iβ* in human *β* cells. 1.1B4 cells were transfected with scrambled siRNA control or STAT3-specific siRNA and then stimulated without or with IL-6 (20 ng/mL) + Dx (100 nM). The mRNA levels of (a)* STAT3*, (b)* REG Iα*, and (c)* REG Iβ* were measured by real-time RT-PCR using *β-actin* as an endogenous control. Data are represented as the ratio of the number of target mRNA copies to the number of *β-actin* mRNA copies and expressed as means ± SE for each group ((a) *N* = 4, (b) *N* = 3-4, (c) *N* = 3). ^*^ *P* < 0.05; ^**^ *P* < 0.0005.

Involvement of STAT3 in the IL-6 + Dx-induced expressions of* REG Iα* and* REG Iβ* was further demonstrated by siRNA. 1.1B4 cells were transfected with scrambled siRNA control or STAT3-specific siRNA and were stimulated with IL-6 + Dx. We confirmed that STAT3-siRNA suppressed both basal and IL-6 + Dx-induced STAT3 mRNA (Figure 6(a)). In the cells treated with the STAT3-siRNA, the IL-6 + Dx-induced upregulation of* REG Iα* and* REG Iβ* was abolished (Figures 6(b) and 6(c)). These results clearly showed the essential requirement of STAT3 in the IL-6 + Dx-induced type I* REG* gene expression in *β* cells.

## 4. Discussion 

*Reg* family genes are involved in cell proliferation and regeneration in several tissues. In rat, type I* Reg* family gene (*Reg I*) is induced during pancreatic *β* cell regeneration [4, 7]. The completion of the human genome sequencing project revealed the complete set of* REG* genes (*REG Iα*,* REG Iβ*,* REG III*,* HIP/PAP*, and* REG IV*) [4, 5, 50, 51]. However, in human, it is not clear which* REG* gene is involved in the *β* cell regeneration. In this study, we found that among five functional human* REG* genes,* REG Iα* and* REG Iβ* gene expressions were significantly enhanced by the combined addition of IL-6 and Dx in human *β* cells. The rat* Reg I* gene expression is activated by IL-6 + Dx [13], and we and others have shown that human REG I*α* protein promotes the proliferation of RINm5F cells and mouse islets [52, 53]. Therefore, in human, as well as in rat [13, 31], the type I REG proteins could be induced under inflammatory conditions, in which the levels of circulating IL-6 and glucocorticoids are increasing and function as a growth factor for *β* cell proliferation.

We demonstrated that IL-6 + Dx induced the type I* REG* gene (*REG Iα* and* REG Iβ*) expression through the JAK/STAT3 pathway. It has been reported that in gastric cancer cells, IL-6 enhanced* REG Iα* transcription through the STAT3 activation [54, 55]. In addition, the enhancement of* REG Iα* promoter activity by IL-6 was reported also in colon cancer cells [56] and in salivary ductal cells [57]. Thus, involvement of STAT3 in* REG Iα* gene expression is not restricted in pancreatic *β* cells but rather common to diverse cell types. However, in *β* cells, Dx is required in addition of IL-6 for the induction, since IL-6 alone only slightly induced* REG Iα* expression. We recently reported that* HGF* gene transcription was also induced by IL-6 + Dx through STAT3 activation in pancreatic *β* cells [31]. It is possible that glucocorticoids are necessary for STAT3 activation in addition to IL-6 in pancreatic *β* cells. The role of glucocorticoids in the* REG Iα* induction is currently under investigation.

The IL-6 + Dx-induced increases, in the promoter activity and the mRNA level of* REG Iα*, were much larger than those of* REG Iβ*. The reason of the difference of inducibility between* REG Iα* and* REG Iβ* is not clear. The expression patterns of two genes are different in pancreas [58]. In addition, we recently reported that, in 1.1B4 cells treated by intermittent hypoxia,* REG Iα* gene expression but not* REG Iβ* gene expression was increased [23]. Thus, although the JAK/STAT pathway seems to be essential to the induction of both type I* REG* genes, these two genes should be regulated independently by other transcription factors under physiological and pathological conditions. Previous [23] and current studies suggest that* REG Iα* gene rather than* REG Iβ* gene should be much more important for *β* cells in the circumstances in which *β* cells are damaged.

IL-22 was reported to induce* REG Iα* transcription via STAT3 activation in human colon cancer cells [59] and to induce* Reg I* and* Reg II* in NOD mouse islets [6, 45, 60]. In addition, Shioya et al. reported IL-22 receptor expression in human pancreatic *β* cells [61]. However, in the present study, either IL-22 alone or IL-22 + Dx only modestly increased the* REG Iα* and* REG Iβ* promoter activities in 1.1B4 cells. Therefore, STAT3 may not be fully activated by IL-22 nor IL-22 + Dx in human pancreatic *β* cells.

In rat RINm5F cells, the cis-element of rat* Reg I* gene for induction in response to IL-6 + Dx was the −81~−70 region [13]. This region in rat* Reg I* promoter corresponds to the −76~−65 region of the human* REG Iα* gene, which is downstream of the STAT-binding site (−142~−134). In reporter assays, the promoter activity of the construct “−130” was not enhanced by the stimulation of IL-6 + Dx. Moreover, the combined addition of 3AB with IL-6 + Dx did not enhance the promoter activity of mutant construct “−146 M” as much as that of “−146.” These results suggest that although PARP was involved in the IL-6 + Dx-induced activation of* REG Iα* promoter, STAT binding to the −142~−134 region played a major role for the induction of* REG Iα* transcription in human *β* cells.

In this study, we found that among five human* REG* family genes, the expressions of* REG Iα* and* REG Iβ* were significantly enhanced by IL-6 + Dx. These results suggest that, under inflammatory conditions, human type I REG proteins could be increased and function as growth factors for *β* cells to facilitate proliferation. Furthermore, we showed that the JAK/STAT pathway was involved in the IL-6 + Dx-induced human type I* REG* gene transcription in *β* cells. Therefore, polymorphisms and/or mutations in* REG Iα* and* REG Iβ* promoters as well as other polymorphisms/mutations of factors involved in the JAK/STAT pathway may be involved in diabetes incidence/pathology.
